# Experience of 5 years adjustable continence therapy (ProACT): the surgical learning curve and patient outcomes

**DOI:** 10.1007/s00345-026-06291-7

**Published:** 2026-03-22

**Authors:** Lisa M. de Zeeuw, Coen H. H. Christiaans, Sebastiaan Remmers, Bertil F. M. Blok, Alexander B. Stillebroer

**Affiliations:** Department of Urology, Erasmus University Medical Center, Rotterdam, The Netherlands

**Keywords:** Postprostatectomy, Urinary incontinence, Success rate, Voiding-frequency diary

## Abstract

**Purpose:**

Post-prostatectomy urinary incontinence (PPI) significantly impacts quality of life. While an artificial urinary sphincter is the standard surgical treatment for PPI, adjustable continence therapy balloons (ProACT) have emerged as a less invasive alternative over the past two decades. ProACT implantation is considered technically challenging and limited to three high-volume Dutch medical centers, yet little is known about the surgical learning curve. This study aims to assess the learning curve to inform training requirements for ProACT implantation.

**Methods:**

This retrospective single-center study included all ProACT implantations performed by one urologist at Erasmus University medical Center, Rotterdam. Surgery success was defined as postoperative use of a maximum of one ‘safety pad’, less than 10 mL urine loss or ≥ 95% subjective improvement of continence. Multivariable logistic regression assessed the learning curve.

**Results:**

A total of 108 surgeries between 2019 and 2024 were included, of which 15 were supervised by an experienced surgeon. No statistical relation was found between the progressive surgeon’s experience and surgical success (per 10 surgeries, OR 0.89, 95%CI: 0.75–1.07, *p* = 0.2). Preoperative incontinence (pads/day) was statistically significantly associated with surgical success (OR 0.65, 95%CI: 0.47–0.89, *p* = 0.009). The overall surgical success rate was 61%, with an additional 20% achieving *>* 50% continence improvement. Complication rate within six months was 21% of which six (5.6%) included explantations of the ProACT device.

**Conclusion:**

ProACT implantation achieves consistent outcomes after brief supervised training, with no statistically significant learning curve. This supports a broader adoption of this technique by urologists treating PPI.

## Introduction

A common complication following radical prostatectomy is postprostatectomy incontinence (PPI), with up to 20% of patients having persistent urine incontinence 12 months after surgery [1, 2]. This complication, to a lesser extent also prevalent after trans-urethral resection of the prostate [3], significantly impacts patients’ quality of life [4, 5]. It is mainly due to sphincter dysfunction as a complication of the surgery and most patients suffer from stress urinary incontinence (SUI) [6]. If initial treatment with pelvic floor muscle therapy fails, surgical intervention is recommended. Of these surgical interventions, an artificial urinary sphincter (AUS) is considered the gold standard, with a 60% cure rate, defined as no use of pads or one ’safety’ pad per day [7, 8, 9].

A different technique treating PPI, using adjustable continence therapy balloons ProACT ^®^, (Uromedica, Inc., Plymouth, MN, USA), was first described in 2005 [10]. In this procedure two silicon balloons are implanted percutaneously through the perineum on either side of the urethra cranial to the pelvic floor. These apply pressure to the urethra and bladder neck, leading to an increased urethral resistance. Once implanted the balloons are filled with fluid and their volume can be adjusted until the optimal effect is reached. A recent meta-analysis of ProACT outcomes reported a surgical success rate of 53%, defined as the use of 0–1 pad per day, and an overall complication rate of 31%, including explantation in 27% of cases [11]. ProACT is less invasive and is performed in a shorter surgical time than AUS [12]. Another significant advantage of ProACT is that, once implanted, it requires no further patient manipulation during urination, unlike AUS, which involves operating a scrotal pump prior to every void [13]. These characteristics make the device a good option for men with impaired hand function and decreased operability. Additionally, in case of problems with the device - such as infection - it can be easily removed in an out-patient setting. Previous studies have shown that efficacy of treating PPI using ProACT is similar to AUS, with fewer complications [14, 15].

Although ProACT implantation is minimally invasive, the surgery is considered technically challenging to master. After its introduction, studies showed that complication and revision rates decreased as surgeons gained more experience [16]. However, no formal evaluation has been done since 2006 to assess the experience required to reduce complications and revisions. Currently only three medical centers in the Netherlands offer this procedure, limiting access for patients at other hospitals. Greater insights into the necessary training and patient outcomes related to surgeon expertise could encourage more centers to adopt this technique.

The primary objective of this study is to map the learning curve of a single surgeon, providing medical centers with insight into the training investment necessary for surgeons to perform this procedure. The secondary objective is to assess post-surgery outcomes, including incontinence, complications and revisions over a five-year period.

## Methods

### Device and procedure

ProACT is a non-circumferential compression device that consists of two separate silicon balloons, attached by tubing to a titanium port [9]. Implantation is performed with the patient in dorsal lithotomy position under general anesthesia. The balloons are implanted through two small incisions in the perineum and placed on either side of the urethra, cranial to the pelvic floor. The two titanium volume adjustment ports are placed subcutaneously in the scrotum through the same incision. After six weeks of healing, patients visit a specialized nurse for volume adjustments. The balloons are filled through the titanium ports with additional contrast fluid in subsequent visits, until the optimal effect of the treatment is achieved, to a maximum of 8 mL. Follow-up in this study ended when patients were discharged because of sufficient effect of treatment or started other treatment for PPI.

### Study design

In this retrospective single-center study, all ProACT implantations performed by one consultant urologist in our center were included up to the end of this study in June 2024 to ensure six months of follow-up. This surgeon (AS) started ProACT implantations in 2019, under supervision of a urologist with over 12 years’ experience in the procedure (BB). We included both surgeries performed under supervision, where the surgeon operated as the primary surgeon, as well as independent procedures. The surgeries where he (AS) observed or assisted as a secondary surgeon, were not included. This study was approved by the local Medical Ethical Review Committee (MEC-2024-0723).

#### Data collection

Data were extracted from outpatient clinic reports, surgical reports and the preoperative urodynamic studies (UDS) of patients with persistent PPI, in whom prior conservative measured had proven insufficient. This included patients with mild or severe PPI, with severe incontinence defined as > 5 pads/day or > 400 g/day (by AUA, undefined by the EAU [17]).

The primary study outcome used to assess the surgical learning curve was incontinence after ProACT implantation, measured in pads/day or a 24-hour pad test, at the timepoint of maximal effect of the ProACT on incontinence. Secondary outcomes included patient reported satisfaction and complications.

Urine loss before and after treatment was measured in patient reported pads per day and 24-hour pad weight tests, kept by patients in a voiding-frequency diary. Postoperative urine loss was assessed at the first follow-up visit after the final volume adjustment, representing the time point of maximal treatment effect. The number of volume adjustments required varied between patients.

Patient-reported subjective improvement was measured during clinic visits by asking patients to rate their improvement since surgery on a 0–100% scale.

Patients were considered ’dry’ when they had a postoperative use of 0 pads or 1 ’safety pad’ per day. When postoperative pads per day was unavailable, a 24-hour urine loss of less than 10 mL or patient-reported subjective improvement more than 95% was also considered ’dry’.

Patients with ’improvement of continence’ are defined as at least 50% reduction in daily pad use or urine loss in mL per day, or a patient reported improvement of at least 50%. All other patients are considered to have no substantial effect of the intervention.

#### Complications

All complications within six months after surgery were scored using the Clavien-Dindo Classification system [18]. Since all patients were seen in the outpatient clinic six weeks postoperatively, most complications were evaluated at this point in time. Earlier complications came to light directly after surgery, or because patients called the urology department. Long-term complications as device failure or erosion after multiple years were not included in our data.

### Statistical methods

The surgical learning curve was analyzed using multivariable logistic regression. A chronological ranking based on date of surgery was applied to all surgeries. The association between this ranking and surgical success was assessed using logistic regression, as described by Vickers et al. [19]. Only unsupervised surgeries were included in the analysis, as supervision could influence surgical outcomes, potentially interfering with the assessment of the learning curve.

Success of surgery was defined in accordance with earlier described definitions. ’Dry’ patients were considered successful, and all other patients were considered unsuccessful. Covariables were preoperative incontinence in pads per day and patient’s age, since incontinence severity has shown to be a negative predictor for ProACT success [20, 21]. Missing data on preoperative pad use per day was imputed five times using multiple imputation by chained equations with the mice package [22]. Restricted cubic splines were used to evaluate non-linear relationship between the number of formerly performed surgeries and the success of surgery [23].

Baseline characteristics were described using median and quartiles for continuous variables and frequency (n) and percentage (%) for categorical variables. Differences in secondary outcomes between observed results (’dry’, ’improved’ or ’no effect’) were described using the Kruskal-Wallis rank sum test for continuous variables and Fisher’s exact test for categorical variables.

A two-sided p-value of less than 0.05 was considered statistically significant. All analyses and figures were generated using R version 4.3.2.

## Results

### Study population

A total of 108 male patients with PPI were included in this study. This includes all surgeries performed by the surgeon AS between September 2019 and June 2024, of which 15 were done under supervision of a specialized surgeon. None of the patients had undergone radiotherapy on the surgical anastomosis, since this is a contraindication for ProACT surgery in our center, because of poor reported outcomes [24, 25].

All baseline characteristics are summarized in Table 1. In four patients the urine loss after the last volume adjustment was not available because they could not be reached for their last telephone appointment, so we used the urine loss assessed at the visit before that.

**Table 1:** Preoperative patient characteristics

		Overall *N* = 108^*1*^	Dry *N* = 66^*1*^	Improved *N* = 22^*1*^	No effect *N* = 20^*1*^
Age, years		73 (70, 76)	72 (69, 75)	76 (71, 78)	73 (70, 76)
BMI, kg/m2		26.3 (24.6, 28.3)	26.2 (24.7, 27.5)	27.0 (24.5, 29.0)	26.7 (23.7, 29.8)
ASA classification	1	9 (8.5%)	7 (11%)	0 (0%)	2 (11%)
	2	66 (62%)	42 (65%)	13 (59%)	11 (58%)
	3	31 (29%)	16 (25%)	9 (41%)	6 (32%)
	*Unknown*	*2*	*1*	*0*	*1*
Prior surgery as cause of SUI	Radical prostatectomy	99 (92%)	60 (91%)	21 (95%)	18 (90%)
	TURP	8 (7.4%)	5 (7.6%)	1 (4.5%)	2 (10%)
	Greenlight laser	1 (0.9%)	1 (1.5%)	0 (0%)	0 (0%)
Preoperative urine loss, pads/day		4.00 (3.00, 5.00)	3.00 (2.50, 5.00)	4.75 (4.00, 6.50)	4.00 (3.00, 5.00)
*Unknown*		*11*	*7*	*4*	*0*
Preoperative urine loss, mL/day		275 (114, 600)	205 (97, 400)	750 (408, 900)	313 (102, 695)
*Unknown*		*47*	*28*	*11*	*8*
Preoperative urgency complaints		48 (44%)	30 (45%)	9 (41%)	9 (45%)
UDS: Bladder capacity, mL		344 (250, 463)	345 (265, 473)	356 (250, 463)	300 (225, 421)
*Unknown*		*17*	*11*	*3*	*3*
UDS: Reduced compliance		23 (26%)	11 (20%)	6 (32%)	6 (35%)
*Unknown*		*18*	*12*	*3*	*3*
UDS: Overactive detrusor		53 (59%)	34 (63%)	12 (63%)	7 (41%)
*Unknown*		*18*	*12*	*3*	*3*
Former treatments	Pelvic floor physiotherapy	81 (75%)	54 (82%)	13 (59%)	14 (70%)
	Neural stimulation	7 (6.5%)	6 (9.1%)	0 (0%)	1 (5.0%)
	Suburethral sling	1 (0.9%)	1 (1.5%)	0 (0%)	0 (0%)
	ProACT	27 (25%)	13 (20%)	7 (32%)	7 (35%)
	Botox	4 (3.7%)	3 (4.5%)	0 (0%)	1 (5.0%)
	AUS	2 (1.9%)	1 (1.5%)	1 (4.5%)	0 (0%)
	None	5 (4.6%)	3 (4.5%)	1 (4.5%)	1 (5.0%)

^*1*^Median (Q1, Q3); n (%)

### Surgical learning curve

**Fig. 1:**
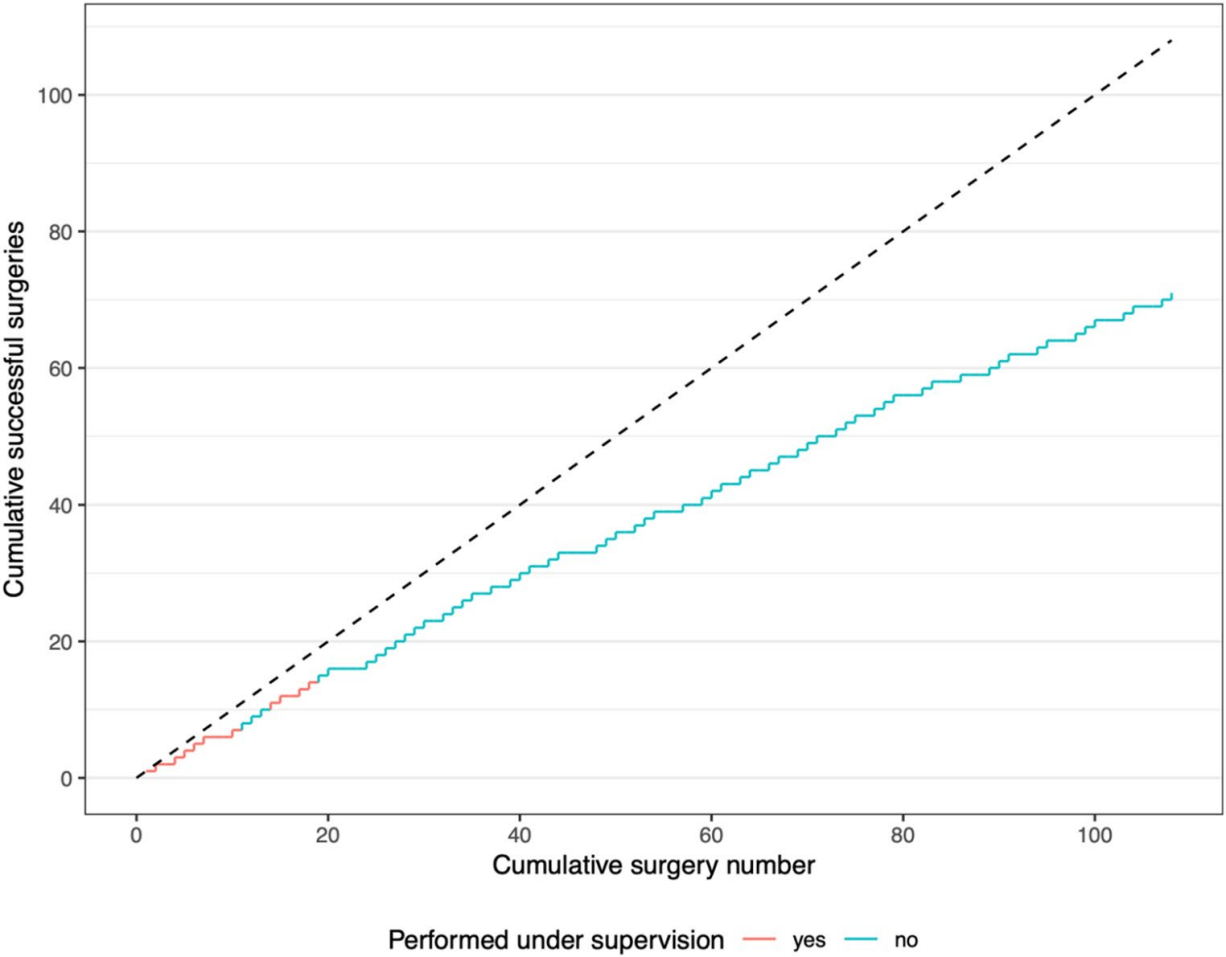
Cumulated sum of successful surgeries over time (with each successful surgery the line inclines with + 1)

In Fig. 1 the cumulated sum of all observed successful surgeries over time is shown. In total, there were 93 unsupervised surgeries, which were included in the logistic regression analysis. Non-linear modelling of the chronological surgery ranking did not improve the fit of the model. There was no statistically significant association between the cumulative number of procedures and surgical success, with an odds ratio per 10 additional procedures of 0.89 (95% CI: 0.75–1.07, *p* = 0.20). Patient age was also not significantly associated with surgical success, with an odds ratio per decade of 0.72 (95% CI: 0.34–1.55, *p* = 0.40). In contrast, preoperative incontinence (measured in pads per day) was significantly associated with surgical success, with an odds ratio of 0.65 (95% CI: 0.47–0.89, *p* = 0.009).

### Efficacy

Results are shown in Table 2. After ProACT implantation and sufficient volume adjustments, 66 patients (61%) were ’dry’ as defined earlier. An additional 22 patients (20%) had at least 50% improvement of continence but were not ‘dry’ and 20 patients (19%) had no effect of the surgery. During 21 surgeries, the in situ malfunctioning ProACT devices were removed, directly prior to implantation of the new device.

Of the patients with little to no effect, 90% underwent other PPI treatment, mostly AUS. Of the patients in whom a 50% reduction of incontinence was reached, 14% had other treatment for their incontinence during our follow-up period.

47% of patients with severe preoperative incontinence reached dryness and a total of 78% had at least 50% improvement.

**Table 2:** Postoperative results, by effect group

	Overall *N* = 108^*1*^	Dry *N* = 66^*1*^	Improved *N* = 22^*1*^	No effect *N* = 20^*1*^	*P*-value^2^
Postoperative urine loss, pads/day	1 (1, 2)	1 (1, 1)	2 (2, 3)	4.75 (4, 6)	< 0.001
*Unknown*	*19*	*4*	*9*	*6*	
Postoperative urine loss, mL/day	19 (4, 70)	5 (1, 13)	58 (46, 250)	430 (242, 750)	< 0.001
*Unknown*	*25*	*15*	*3*	*7*	
Subjective improvement of incontinence	90 (55, 95)	95 (90, 95)	80 (60, 95)	0 (0, 0)	< 0.001
*Unknown*	*57*	*41*	*4*	*12*	
Operating time	25 (20, 30)	24 (18, 29)	26 (23, 32)	29 (22, 34)	0.062
*Unknown*	*6*	*4*	*2*	*0*	
Proact in situ removed during surgery	21 (19%)	9 (14%)	7 (32%)	5 (25%)	0.14
Performed under supervision	15 (14%)	10 (15%)	2 (9.1%)	3 (15%)	0.8
Number of postoperative outpatient visits	6 (4, 8)	4 (3, 6)	8 (5, 11)	8 (6, 12)	< 0.001
Total volume filled into ProACT balloons	4.0 (2.1, 5.8)	3.0 (2.0, 4.0)	5.3 (4.0, 7.1)	7.3 (4.0, 8.0)	< 0.001
Undergone other treatment	21 (19%)	0 (0%)	3 (14%)	18 (90%)	< 0.001
Follow-up, months	7 (5, 12)	6 (5, 12)	9 (6, 14)	6 (5, 9)	0.05
^*1*^Median (Q1, Q3); n (%)					

^*2*^Kruskal-Wallis rank sum test; Fisher’s exact test

### Complications

A total complication rate of 21% within the first six months (182 days) after surgery was observed, see Table 3. This included 5 (4.6%) complications of Clavien-Dindo grade I, 12 (11%) of grade II and 6 (5.6%) of grade III. Intraoperative perforations were treated with a catheter left for 1 week and antibiotics while the ProACT implantation was finished as planned. In three of the patients where one balloon was explanted, more than 50% improvement was reached by increasing the volume of the contralateral balloon. One patient achieved dryness with one balloon.

**Table 3:** Complications and their Clavien-Dindo classification

	*N* = 108	Cause	Management
**No complication**	88 (81%)		
**Grade I**	5 (4.6%)	Leaking balloon (1), yeast infection (1), postoperative urinary retention (2), hematoma (1)	No intervention, indwelling catheter
**Grade II**	12 (11%)	Intraoperative bladder/urethra perforation (3), infection of ProACT system (3), wound infection (3), hematoma (1), epididymitis (1), fausse route catheter (1)	Antibiotics
**Grade III**	6 (5.6%)	Infection of ProACT system (2), persistent scrotal/perineal pain (3), erosion (1)	Explantation of ProACT system

## Discussion

In the last two decades, ProACT device implantations have evolved as a surgical technique in treating PPI. It is widely established that surgical outcomes improve as a surgeon gains experience [26], a principle supported by earlier studies on surgical learning curves [27]. However, limited research has been done on the surgical learning curve of the ProACT implantation since Hübner et al. reported their findings in 2006 [16].

### Learning curve analysis

We found no statistically significant effect of surgical experience over a 5-year time period on surgical success rates, defined by postoperative continence. This suggests that for a consultant urologist, a supervised training period of approximately 15 surgeries is sufficient to achieve satisfactory operative results.

The observed absence of a learning curve differs from earlier results reported by Hübner et al. [16], who observed better results in their latter cohort.

One key difference may be that Hübner et al. conducted their research shortly after the introduction of ProACT, when surgical techniques and postoperative protocols were still evolving. During the current study, no such updates were made. Moreover, the surgeon in the current study was trained by a urologist highly proficient in ProACT implantation.

In some learning curve studies, operating time is used as an indicator of surgical proficiency. However, in ProACT implantations, the relatively short surgical time is influenced by factors beyond the surgeon’s skill, such as the experience of the surgical team and whether malfunctioning ProACT devices were simultaneously removed during surgery. While we observed a statistically significant difference in surgery times between the three outcome groups, we consider this difference clinically irrelevant. Notably, the ’dry’ group, with the shortest surgical time, also had less ProACT removals, which could explain this difference.

### Efficacy and safety

Overall efficacy of ProACT in this study aligns with prior research, with 61% of patients achieving dryness and 81% achieving at least 50% improvement of continence. The most recent meta-analysis on this subject reported an overall dryness rate of 55%, with varying definitions of dryness across studies [11].

We only reported complications occurring in the first 6 months post-surgery, as this was the follow-up period available for all the patients included, resulting in a complication rate of 21%. In the literature, complication rates vary between 12% and 31% in meta-analyses [11, 14]. In our cohort, the intraoperative urethra/bladder perforation rate was 2.8%, lower than the meta-analyses estimate of 5.3% [15]. This supports the absence of a significant learning curve in this dataset, as it includes the first ProACT implantation surgeries performed by a surgeon and does not fall short of the outcomes reported in the literature.

### Predictors of surgical success

Previous studies have shown that severe preoperative incontinence is a negative predictor for postoperative dryness [20, 21]. Our results confirm this, where a significant correlation between pre- and postoperative incontinence is noted. Our findings highlight the importance of patient selection and counseling. Instead of focusing on surgical experience, more attention should be given to identify preoperative predictors of success to optimize procedure outcomes.

However, ProACT can still be a viable option for patients with severe incontinence, since 47% of our patients with severe incontinence achieved dryness and a total of 78% experienced at least a 50% improvement in symptoms. In our study, only 14% of the patients who experienced a 50% reduction in incontinence (‘improved group’) had another treatment for their incontinence, opposed to 90% of patients who had no effect of the surgery. In this ’improved’ group, we observe a median subjective improvement of 80%. This indicates that, while this group does not achieve ’dryness’, they still have a clinically relevant effect of the ProACT implantation.

Reduced bladder compliance and detrusor overactivity are also highly correlated to PPI and most PPI patients do not have sheer stress incontinence, but also an urgency-incontinence component [28]. It has also been shown that successful ProACT treatment decreases bladder contractility parameters [20, 29]. In this study the UDS results showed reduced compliance in 26% of patients and detrusor overactivity in 59%. We recommend prospective research into preoperative predictors of success to optimize ProACT outcomes.

### Study limitations

There are some limitations to this study. First, there was a limited duration of follow-up since our most recent patients were treated in 2024. Because of this, we were not able to report on long-term results of the ProACT device. A second limitation is that incontinence was primarily measured using patient-reported pad usage, as not all patients completed incontinence diaries. While pad usage is a widely accepted measure of continence [30], it is not standardized. Studies on the correlation between pad usage and actual urine loss in grams have yielded mixed results, with some showing strong correlation [31] and others recommending against its use for objective measurement [32]. At last, the analyzed learning curve only included one surgeon. It is plausible that interpersonal variation plays a role in learning curves. However, given that no clear trend is observed in the current data and the observed efficacy is comparable to results in previous literature, it seems unlikely that such variation would manifest in a significant learning curve in a larger group of surgeons. This is particularly true since the ProACT procedure is often performed by experienced urologists who are incorporating this specific intervention into their existing practice.

## Conclusions

Our findings demonstrate that ProACT implantation achieves consistent results after a short, supervised training period, with no evidence of a significant learning curve. This supports a broader adoption of ProACT as a treatment option by urologists who treat PPI. We advise a training period supervised by a urologist sufficient in ProACT implantations. Additionally, our study emphasizes the importance of patient selection and highlights the need for further research into preoperative predictors to optimize surgical outcomes and patient satisfaction.

## Data Availability

The data supporting the findings of this study are not publicly available due to sensitivity and patient privacy concerns. However, they can be obtained from the corresponding author upon reasonable request. Data are located in controlled access data storage at Erasmus University Medical Center.

## References

[1] Buckley BS, Lapitan MCM, Epidemiology Committee of the Fourth International Consultation on Incontinence (2008), Paris, Prevalence of urinary incontinence in men, women, and children–current evidence: findings of the Fourth International Consultation on Incontinence. Urology. 2010 Aug;76(2):265 – 70. 10.1016/j.urology.2009.11.078 10.1016/j.urology.2009.11.078 20541241

[2] Haglind E, Carlsson S, Stranne J, Wallerstedt A, Wilderäng U, Thorsteinsdottir T et al (2015) Urinary incontinence and erectile dysfunction after robotic versus open radical prostatectomy: A Prospective, Controlled, nonrandomised trial. Eur Urol 68(2):216–225. 10.1016/j.eururo.2015.02.029 25770484 10.1016/j.eururo.2015.02.029

[3] Rassweiler J, Teber D, Kuntz R, Hofmann R (2006) Complications of transurethral resection of the prostate (TURP)–incidence, management, and prevention. Eur Urol 50(5):969–979 discussion 980. 10.1016/j.eururo.2005.12.042 16469429 10.1016/j.eururo.2005.12.042

[4] Buckley BS, Lapitan MCM, Glazener CM, MAPS Trial Group. The effect of urinary incontinence on health utility and health-related quality of life in men following prostate surgery. Neurourol Urodyn 2012 Apr;31(4):465–469. 10.1002/nau.21231 10.1002/nau.21231 22396387

[5] Köhler N, Friedrich M, Gansera L, Holze S, Thiel R, Roth S et al (2014 Nov) Psychological distress and adjustment to disease in patients before and after radical prostatectomy. Results of a prospective multi-centre study. Eur J Cancer Care (Engl) 23(6):795–802. 10.1111/ecc.12186 10.1111/ecc.12186 24661440

[6] Singla AK (2007 Apr) Male incontinence: pathophysiology and management. Indian J Urol 23(2):174–179. 10.4103/0970-1591.32070 10.4103/0970-1591.32070 PMC2721528 19675796

[7] Gacci M, Sakalis VI, Karavitakis M, Cornu JN, Gratzke C, Herrmann TRW et al (2022) European association of urology guidelines on male urinary incontinence. Eur Urol 82(4):387–398. 10.1016/j.eururo.2022.05.012 35697561 10.1016/j.eururo.2022.05.012

[8] Chen YC, Lin PH, Jou YY, Lin VCH (2017 May) Surgical treatment for urinary incontinence after prostatectomy: A meta-analysis and systematic review. PLoS ONE 12(5):e0130867. 10.1371/journal.pone.0130867 10.1371/journal.pone.0130867 PMC5415174 28467435

[9] Lucas MG, Bosch RJL, Burkhard FC, Cruz F, Madden TB, Nambiar AK et al (2012) EAU guidelines on surgical treatment of urinary incontinence. Eur Urol 62(6):1118–1129. 10.1016/j.eururo.2012.09.023 23040204 10.1016/j.eururo.2012.09.023

[10] Hübner WA, Schlarp OM (2005 Sep) Treatment of incontinence after prostatectomy using a new minimally invasive device: adjustable continence therapy. BJU Int 96(4):587–594. 10.1111/j.1464-410X.2005.05689.x 10.1111/j.1464-410X.2005.05689.x 16104915

[11] Tricard T, Song QX, Munier P, Li JY, Leng J, Saussine C et al (2023) Adjustable continence therapy (proACT) for the treatment of male stress urinary incontinence post-prostatectomy: a systematic review and meta-analysis (2023 update). World J Urol 41(7):1793–1802. 10.1007/s00345-023-04452-6 37311990 10.1007/s00345-023-04452-6

[12] Martens FMJ, Lampe MI, Heesakkers JPFA (2009) ProACT for stress urinary incontinence after radical prostatectomy. Urol Int 82(4):394–398. 10.1159/000218526 19506404 10.1159/000218526

[13] den Hoedt S, Blok BFM (2024 Aug) Adjustable continence therapy (ProACT/ACTTM) with periurethral balloons for treatment of stress urinary incontinence: a narrative review. Transl Androl Urol 13(8):1744–1761. 10.21037/tau-22-807 10.21037/tau-22-807 PMC11399041 39280649

[14] Crivellaro S, Morlacco A, Bodo G, Agro’ EF, Gozzi C, Pistolesi D et al (2016) Systematic review of surgical treatment of post radical prostatectomy stress urinary incontinence. Neurourol Urodyn 35(8):875–881. 10.1002/nau.22873 26397171 10.1002/nau.22873

[15] Larson T, Jhaveri H, Yeung LL (2019) Adjustable continence therapy (ProACT) for the treatment of male stress urinary incontinence: A systematic review and meta-analysis. Neurourol Urodyn 38(8):2051–2059. 10.1002/nau.24135 31429982 10.1002/nau.24135

[16] Hübner WA, Schlarp OM Adjustable continence therapy (ProACT): evolution of the surgical technique and comparison of the original 50 patients with the most recent 50 patients at a single centre. Eur Urol 2007 Sep;52(3):680–686. 10.1016/j.eururo.2006.10.054 10.1016/j.eururo.2006.10.054 17097218

[17] Bhatt NR, Pavithran A, Ilie C, Smith L, Doherty R (2024 May) Post-prostatectomy incontinence: a guideline of guidelines. BJU Int 133(5):513–523. 10.1111/bju.16233 10.1111/bju.16233 38009420

[18] Dindo D, Demartines N, Clavien PA (2004 Aug) Classification of surgical complications: a new proposal with evaluation in a cohort of 6336 patients and results of a survey. Ann Surg 240(2):205–213. 10.1097/01.sla.0000133083.54934.ae 10.1097/01.sla.0000133083.54934.ae PMC1360123 15273542

[19] Vickers AJ, Cronin AM, Masterson TA, Eastham JA (2010) How do you tell whether a change in surgical technique leads to a change in outcome? J Urol 183(4):1510–1514. 10.1016/j.juro.2009.12.034 20172569 10.1016/j.juro.2009.12.034 PMC2859681

[20] Utomo E, Groen J, Vroom IH, van Mastrigt R, Blok BFM (2013 Jun) Urodynamic effects of volume-adjustable balloons for treatment of postprostatectomy urinary incontinence. Urology 81(6):1308–1314. 10.1016/j.urology.2013.01.020 10.1016/j.urology.2013.01.020 23465144

[21] Noordhoff TC, Scheepe JR, Blok BFM (2018) Outcome and complications of adjustable continence therapy (ProACT™) after radical prostatectomy: 10 years’ experience in 143 patients. Neurourol Urodyn 37(4):1419–1425. 10.1002/nau.23463 29266406 10.1002/nau.23463

[22] van Buuren S, Groothuis-Oudshoorn K (2011) Mice: multivariate imputation by chained equations in R. J Stat Soft 45(3):1–67

[23] Harrell FE Jr (2024) rms: Regression Modeling Strategies; R package version 6.8-0. Available from: https://CRAN.R-project.org/package=rms

[24] Finazzi Agr`o E, Gregori A, Bianchi D, Carone R, Giamm`o A, Ammirati E et al (2019 Sep) Efficacy and safety of adjustable balloons (Proact™) to treat male stress urinary incontinence after prostate surgery: medium and long-term follow-up data of a National multicentric retrospective study. Neurourol Urodyn 38(7):1979–1984. 10.1002/nau.24103 10.1002/nau.24103 31302928

[25] Lebret T, Cour F, Benchetrit J, Grise P, Bernstein J, Delaporte V et al (2008) Treatment of postprostatectomy stress urinary incontinence using a minimally invasive adjustable continence balloon device, proact: results of a preliminary, multicenter, pilot study. Urology 71(2):256–260. 10.1016/j.urology.2007.08.062 18308096 10.1016/j.urology.2007.08.062

[26] Khan N, Abboudi H, Khan MS, Dasgupta P, Ahmed K (2014 Mar) Measuring the surgical ’learning curve’: methods, variables and competency. BJU Int 113(3):504–508. 10.1111/bju.12197 10.1111/bju.12197 23819461

[27] Cook JA, Ramsay CR, Fayers P (2004) Statistical evaluation of learning curve effects in surgical trials. Clin Trials 1(5):421–427. 10.1191/1740774504cn042oa 16279280 10.1191/1740774504cn042oa

[28] Leach GE, Trockman B, Wong A, Hamilton J, Haab F, Zimmern PE (1996 Apr) Post-prostatectomy incontinence: urodynamic findings and treatment outcomes. J Urol 155(4):1256–1259. 10.1016/s0022-5347(01)66235-9 10.1016/s0022-5347(01)66235-9 8632545

[29] Reuvers SHM, Groen J, Scheepe JR, Blok BFM Maximum urethral closure pressure increases after successful adjustable continence therapy (ProACT) for stress urinary incontinence after radical prostatectomy. Urol 2016 Aug;94:188–192. 10.1016/j.urology.2016.04.019 10.1016/j.urology.2016.04.019 27130261

[30] Neto WA, Capibaribe DM, Dal Col LSB, Andrade DL, Moretti TBC, Reis LO (2022 May) Incontinence after laparoscopic radical prostatectomy: a reverse systematic review. Int Braz J Urol 48(3):389–396. 10.1590/S1677-5538.IBJU.2021.0632 10.1590/S1677-5538.IBJU.2021.0632 PMC9060170 35168312

[31] Nitti VW, Mourtzinos A, Brucker BM, SUFU Pad Test Study Group (2014) Correlation of patient perception of pad use with objective degree of incontinence measured by pad test in men with post-prostatectomy incontinence: the SUFU pad test study. J Urol 192(3):836–842. 10.1016/j.juro.2014.03.031 24650425 10.1016/j.juro.2014.03.031

[32] Sacco E, Bientinesi R, Gandi C, Di Gianfrancesco L, Pierconti F, Racioppi M et al (2019 May) Patient pad count is a poor measure of urinary incontinence compared with 48-h pad test: results of a large-scale multicentre study. BJU Int 123(5A):E69–78. 10.1111/bju.14566 10.1111/bju.14566 30253042

